# Annotations

**Published:** 1901-05-18

**Authors:** 


### Annotations.

### Hereditary Inebriety.

The report presented to the Society for the Study of Inebriety by a special committee appointed "to investigate the conditions under which the tendency to inebriety is capable of transmission to offspring" is an interesting document, and is rendered all the more so by the fierce criticisms of its methods and conclusion appended by some of those who signed it. The report says that "the genesis of inebriety in the individual depends on three essential factors, of which one is inborn and the others acquired." The inborn factor is stated to be "a capacity for enjoying the sensations evoked by alcohol," while the acquired factors are "a personal experience of the sensations evoked by alcohol" and "the increased delight in drink which continued indulgence in drink confers." This distinction is then drawn: "The inborn capacity for enjoying alcohol, like other inborn traits, is certainly heritable, and for this reason, among others, it is that one drunken generation succeeds another. On the other hand there is no evidence that acquired characters are heritable. In particular there is no evidence that characters acquired by the parent through indulgence in drink are inherited by the children subsequently born." Probably the committee was torn between the desire to explain the facts as they appear to the plain man, who only takes cognisance of one or two generations, and a craving to be "scientific" and to speak in accordance with the formulæ of Weismann. The result is that the report loses much in force. Another side of the question is well put in the separate report signed by Mr. Henry Kesteven, who, instead of dealing with inebriety as a something separate and all to itself—"a capacity for enjoying the sensations evoked by indulgence in alcohol,"—lumps it with other forms of lack of self-control, which may be inborn or may be acquired by the individual. He says that the craving "for the gratification of the animal sensations is inborn in the race," and he presupposes a "power of self-control" by which this craving is kept in order. Again, he holds that the toxic action of the excessive use of alcohol not only impairs the nutrition metabolism of the somatic tissues, producing degenerative changes, but also "taints and diminishes the vitality of the germinal tissues," the lesion so produced showing itself in the offspring developing from such germinal tissues by affections of those nervous elements which are the latest products of evolution, and therefore the least stable, which also constitute the basis of the higher mental operations. This condition may manifest itself in various ways, among others "in a diminution from the average mental power of self-control," and it is under this that the tendency to inebriety is met with. But what is self-control? Some look upon man as a machine with self-indulgent, and mostly degrading tendencies, ruled by his "will," a power of sel-fcontrol which they regard as his highest attribute; while others look upon man, his tendencies, and his power of controlling them as one and indivisible, the character of the whole being determined by the germ plasm around which his somatic tissues have taken form. Again, as regards this germ plasm some look upon it as capable, like other tissue, of being altered by its environments, while others seek for the cause of every apparent variation in some preceding variation during antecedent generations. On the very bases of the argument there is no agreement.

### County "Isolation" Hospitals.

The Isolation Hospitals Bill now before Parliament has for its object among other things to enable a local authority which has provided "a hospital for the reception of the sick," under the Act of 1875 or any local Act, to transfer such hospital to the county council for use by a "district formed under the Isolation Hospitals Act, 1893," as an isolation hospital. This is a matter of considerable interest in regard to the uses to which such a hospital may be put. The Act of 1875 authorises a sanitary authority to provide for the use of the inhabitants of their district hospitals for the reception of the sick, without any definite limitation in regard to the nature of the sickness, and already certain authorities have shown a desire to extend the meaning of this clause so as to embrace other than infectious diseases. Not every authority, however, is likely to care to take upon itself the responsibility of maintaining at its own expense a general hospital for the treatment of all kinds of diseases. But the matter might be somewhat different should the maintenance of these hospitals be transferred to the county councils. According to the wording of the new Bill it would seem that the hospitals so transferred must be used for "isolation," however that word may be defined; but should any district (from its position far from other hospitals or other cause) be able to persuade the county authority to permit the use of the county council hospital for non-infectious diseases, it would be difficult to prevent other contributing districts claiming the same privilege. Unless safeguards are introduced in regard to the use to which these hospitals are put, this Bill may prove the thin end of a very large wedge the operation of which would certainly be injurious to the best interests of the hospitals of the country, although if kept to its proper purposes it seems likely to be of great utility.

---

### Hospitals as Hot-beds of Infection.

Those who maintain that our modern isolation hospitals are guilty of creating a new type of scarlet fever of such infectivity as to cause the disease to prevail even more in "isolation" towns than it does where no hospitals exist will probably find it difficult to explain a fact lately stated by Mr. Walter Long in the House of Commons. Dr. Farquharson had asked whether his attention had been called to the fact that 47 children had been removed from the Forest Gate School to the fever hospitals of the Metropolitan Asylums Board under the erroneous belief that they were suffering from scarlet fever, and whether any of the children had contracted scarlet fever when in the fever hospitals. To this Mr. Long replied that the fact was as stated; that, following some undoubted cases of scarlet fever, a number of suspicious cases occurred which were sent to the fever hospital as being affected with scarlet fever but ultimately turned out not to be suffering from that disease. The interesting point, however, is that he further stated that none of these children contracted scarlet fever as the result of their adventure. This does not look like the fever wards of our isolation hospitals having become so charged with infection of increased virulence and infective power as to be dangerous to the country.

